# Reliability of the Balance Evaluation Systems Test (BESTest) and BESTest sections for adults with hemiparesis

**DOI:** 10.1590/bjpt-rbf.2014.0033

**Published:** 2014

**Authors:** Letícia C. Rodrigues, Aline P. Marques, Paula B. Barros, Stella M. Michaelsen

**Affiliations:** 1 Graduate Program in Human Movement Sciences, Health Sciences and Sports Center, Universidade do Estado de Santa Catarina (UDESC), Florianópolis, SC, Brazil; 2 Physical Therapy Department, Health Sciences and Sports Center, UDESC, Florianópolis, SC, Brazil; 3 Master's Program in Physical Therapy, UDESC, Florianópolis, SC, Brazil

**Keywords:** stroke, psychometric properties, outcome, rehabilitation

## Abstract

**BACKGROUND:**

The Balance Evaluation Systems Test (BESTest) was recently created to allow the development of treatments according to the specific balance system affected in each patient. The Brazilian version of the BESTest has not been specifically tested after stroke.

**OBJECTIVE:**

To evaluate the intra- and inter-rater reliability and concurrent and convergent validity of the total score of the BESTest and BESTest sections for adults with hemiparesis after stroke.

**METHOD:**

The study included 16 subjects (61.1±7.5 years) with chronic hemiparesis (54.5±43.5 months after stroke). The BESTest was administered by two raters in the same week and one of the raters repeated the test after a one-week interval. Intraclass correlation coefficient (ICC) was calculated to assess intra- and interrater reliability. Concurrent validity with the Berg Balance Scale (BBS) and convergent validity with the Activities-specific Balance Confidence scale (ABC-Brazil) were assessed using Pearson's correlation coefficient.

**RESULTS:**

Both the BESTest total score (ICC=0.98) and the BESTest sections (ICC between 0.85 and 0.96) have excellent intrarater reliability. Interrater reliability for the total score was excellent (ICC=0.93) and, for the sections, it ranged between 0.71 and 0.94. The correlation coefficient between the BESTest and the BBS and ABC-Brazil were 0.78 and 0.59, respectively.

**CONCLUSIONS:**

The Brazilian version of the BESTest demonstrated adequate reliability when measured by sections and could identify what balance system was affected in patients after stroke. Concurrent validity was excellent with the BBS total score and good to excellent with the sections. The total scores but not the sections present adequate convergent validity with the ABC-Brazil. However, other psychometric properties should be further investigated.

## Introduction

Hemiparesis after stroke often results in deficits in balance and risk of falls. In these patients, balance stability is generally decreased due to muscle weakness, sensory loss, and visuospatial impairments. The maintenance of static and dynamic balance stability involves activity integration of the sensory and motor systems, usually affected in these individuals^1^ ^-^ ^3^.

Balance impairments are associated with lower ambulatory activity, physical deconditioning, and high risk of falls in this population^4^ ^,^ ^5^, affecting the performance of many activities of daily living^6^. Therefore, balance assessment is important for the proper prescription of auxiliary devices, development of treatment interventions, and identification of safe and unsafe activities for people with stroke. Consequently, it is important that clinicians have reliable measures to detect changes that occur during the rehabilitation process^7^.

The Berg Balance Scale (BBS) was created to assess the risk of falls in the elderly. It has been the main tool used to identify and evaluate balance impairment in different populations^8^, however it has floor and ceiling effects and therefore cannot detect significant changes when used to assess stroke patients with severe or mild impairment, respectively^7^ ^,^ ^9^.

The balance section of the Fulg-Meyer Scale evaluates some limitations in performing tasks involving change in body position and maintenance^10^. Just as the BBS, this section may not be appropriate for use in patients who are severely affected or to detect improvement in those who are slightly affected initially^11^.

In addition to balance performance, confidence in performing tasks without falling is essential to preserving autonomy in daily activities. The Activities-specific Balance Confidence scale (ABC) was developed to numerically quantify the level of confidence in balance and essentially evaluate a person's perception of their ability to perform specific activities without falling or becoming unsteady^12^. The ABC has good accuracy to detect people with stroke with a history of multiple falls^13^.

The standardized clinical tools for assessing balance can predict the risk of falls, especially in elderly people, but in general they cannot identify which balance system has been affected. Therefore, the Balance Evaluation Systems Test (BESTest) was developed in 2009. It consists of 27 tasks (items), divided into six sections, grouped in order to reveal the function or dysfunction of a specific balance control system (biomechanical constraints, stability limits/verticality, anticipatory postural adjustments, postural responses, sensory orientation, and stability in gait). Identifying which balance control system is impaired helps to specifically direct the treatment^14^. This scale is used to assess balance in elderly individuals and various diseases, but it has not been specifically validated for people with stroke. The study by Tsang et al.^15^ examined the reliability and validity of the English version of the Mini-BESTest in people with hemiparesis, but this short version of the BESTest excluded sections I and II, i.e. Biomechanical Constraints and Stability limits/Verticality, respectively. Section I, for example, evaluates base of support and hip and ankle strength and section II, lateral lean as well as functional reaching. We hypothesize that those items may be important to assess in individuals with hemiparesis.

The BESTest and Mini-BESTest were translated and cross-culturally adapted to Brazilian Portuguese by Maia et al.^16^ and their reliability was assessed in elderly people and individuals with Parkinson's Disease. The reliability and validity of the translated version have not been evaluated in patients with hemiparesis. This group has particular characteristics that differ from other populations, therefore it is important and necessary to adapt some tasks of the BESTest for this group. Thus, the aim of this study was to evaluate the intra- and interrarter reliability and concurrent and convergent validity of the BESTest and BESTest sections for adults with hemiparesis.

## Method

This study obtained ethical approval from the Human Research Ethics Committee (ETIC 227/2010) of Universidade do Estado de Santa Catarina (UDESC), Florianópolis, SC, Brazil.

### Adaptation of the administration of the Brazilian version of the BESTest16 for adults with hemiparesis

A translated version was produced by our research group and then a Brazilian version was published by Maia et al.^16^. A committee of practicing physical therapists analyzed both versions, and because only minor differences were detected, we decided to adopt the published version and only adapt the administration of this version for the study population. The adaptations were based on differences presented by individuals with hemiparesis and are adopted to standardize the form of administration. All adaptations were decided in conjuction by the committee.

### Participants

This study included 16 adult and elderly patients (49 to 73 years old) with chronic hemiparesis (3 to 150 months after stroke) recruited from the UDESC physical therapy clinic and extension programs (Table 1).

**Table 1.** Participants characteristics.

	Mean (SD)
Age (years)	61.1 (7.5)
Gender (M/F)	13M/3F
Time of stroke (months)	54.5 (43.5)
Paretic side (R/L)	3R/13L
Dominant side (R/L)	16R/0L
ABC-Brazil (%)	43.7 (22.0)
BBS (score)	48.6 (5.4)
Mini-Mental	26.6 (3.1)

SD = standard deviation

M =male

F =female

R =right

L =left

**ABC-Brazil:** =Brazilian version of the Activities-specific Balance Confidence Scale

BBS = Berg Balance Scale.

We included individuals who met the following inclusion criteria: hemiparesis resulting from stroke affecting one of the cerebral hemispheres, ability to understand the instructions (cutoff score according to educational level on the Mini-Mental State Examination)^17^ ^,^ ^18^, and ability to stand confidently without assistance for at least 2 minutes. We excluded patients with other neurological diseases and cerebellar stroke. All participants signed an informed consent form to participate in this study.

### Intra- and interrater reliability of the BESTest-Brazil

The interrater reliability was tested by comparing the evaluations of two independent raters (R1 and R2) who conducted the assessment on the same day. The intrarater reliability (test-retest) was tested by the same rater by comparing the results of repeated assessments (R1T1 and R1T2) with a one-week interval.

### Convergent and concurrent validity

The convergent and concurrent validity of the Brazilian version of the BESTest was assessed with the ABC-Brazil and the BBS. The ABC-Brazil contains 16 questions and measures the balance confidence of individuals while performing specific activities, including tasks outside home. The response is evaluated by a visual analog scale ranging from 0 to 100, with 0=no confidence and 100=complete confidence^19^.

The Brazilian version of the BBS consists of 14 items that assess static and dynamic balance. Each item is scored on a five-point scale from 0 (unable to perform) to 4 (normal performance). The highest possible score is 56, with higher scores indicating better balance^8^.

### Statistical analysis

We used intraclass correlation coefficient (ICC) and a confidence interval (CI) of 95% to evaluate intrarater (test-retest) and interrater reliability. The following classification was used for the ICC values: poor reliability ICC<0.40, moderate reliability ICC<0.75, and excellent reliability ICC>0.75^20^.

The convergent and concurrent validity of the scores of each session and total score of the BESTest with the total BBS and ABC-Brazil was assessed using Pearson's correlation coefficient. The following classification was used for correlation: <0.49 poor, 0.50 to 0.69 moderate, and >0.70 strong^21^.

## Results

To consider the specificity of the individuals with hemiparesis, the expert committee adopted some adaptations in the form of administering the BESTest (Table 2). In the instructions for administration of the original version of the BESTest^14^, item 2 considers abnormal segmental postural alignment such as scoliosis or kyphosis or asymmetries. Our adapted version considers the paretic upper limb abnormal alignment because it is frequent in individuals with hemiparesis. In item 7, the patient is instructed to reach forward with both arms straight without touching the ruler or the wall. In our adapted version, the non-paretic upper limb was positioned near the wall because it is difficult for patients to raise their paretic arms in the position recommended by the test. In items 10, 11, 12, 19, and 20 of the original version, the patients are asked to place hands on hips, but in our version they were allowed to place their arms along the body because almost all patients were unable to do this with their paretic limb due to upper limb paresis and absence of distal movements. Finally, in item 13 of the original version, the task required was to lift a weight with both hands up to shoulder level, however patients with hemiparesis had difficulty lifting the weight with their paretic upper limb, so they were allowed to lift it only with the non-paretic upper limb and decrease the weight if necessary.

**Table 2.** Adaptations to the BESTest sections for administration in adults with hemiparesis.

Section	Item	Adaptation
I – Biomechanical Constraints	2. CoM alignment	Upper limb posture was considered in abnormal segmental postural alignment
II – Stability limits/ verticality	7. Functional reach forward	The non-paretic upper limb was positioned near the wall
III – Anticipatory postural adjustments	10. Rise to toes	If patients were unable to keep hands on hips, they could complete the test with arms along the body.
	11. Stand on one leg	If patients were unable to keep hands on hips, they could complete the test with arms along the body.
	12. Alternate stair touching	If patients were unable to keep hands on hips, they could complete the test with arms along the body.
	13. Standing arm raise	If patients were unable to lift weight with both hands, they could lift the weight only with the non-paretic hand and decrease the weight.
V - Sensory Orientation	19. Sensory integration for balance (modi?edCTSIB)	If patients were unable to keep hands on hips, they could complete the test with arms along the body.
	20. Incline—eyes closed	If patients were unable to keep hands on hips, they could complete the test with arms along the body.

Among the BESTest sections, the participants with hemiparesis showed a better performance in Sensory Orientation*.* Most participants achieved the maximum score on all items in this section, and item 19D (Eyes closed, foam surface) was the only in which some participants had difficulties. The lowest scores were in the sections Biomechanical Constraints and Anticipatory Postural Adjustments (Table 3). In these sections, the lower scores were in item 4 (Hip/trunk lateral strength) and item 11 (stand on one leg), respectively.

**Table 3.** Descriptive Statistics and intra- and interrater reliability of the sections and total score of the BESTest in a sample of subjects with hemiparesis.

Section	R1_T1Mean(SD)	R1_T2Mean(SD)	ICC(CI 95%)Intra-R	R2Mean(SD)	ICC(CI 95%)Inter-R
Section I: Biomechanical Constraints (%)	46.7(21.9)	46.7 (21.3)	0.95(0.85-0.98)	62.1 (21.9)	0.94(0.83-0.98)
Section II: Stability limits/verticality (%)	71.4 (10.6)	70.6(9.7)	0.86(0.64-0.96)	75.7 (7.9)	0.74(0.25-0.91)
Section III: Anticipatory postural adjustments (%)	51.7(18.7)	54.9(16.5)	0.90(0.71-0.96)	58.2(16.7)	0.88(0.66-0.96)
Section IV: Postural responses (%)	66.0 (19.1)	72.8(22.6)	0.85(0.58-0.95)	84.4(16.6)	0.72(0.20-0.90)
Section V: Sensory Orientation (%)	74.6 (16.9)	72.9 (17.6)	0.87(0.68-0.96)	90.0(12.9)	0.71(0.16-0.90)
Section VI: Stability in gait (%)	54.5 (21.0)	54.4 (20.1)	0.96(0.89-0.99)	68.3(18.5)	0.80(0.43-0.93)
**Total score **(%)	60.4(14.9)	61.6(14.8)	0.98(0.94-0.99)	73.1(12.6)	0.93(0.80-0.98)

R1 =rater 1

T1 =test 1

T2 =test 2

R2 =rater2

SD =standard deviation

ICC =intraclass correlation coefficient

CI =confidence interval of 95%

The intrarater reliability was excellent (between 0.85 and 0.98) in all sections and in the total score. The interrater reliability was also excellent for sections I, III, VI, and the total score, and moderate for sections II, IV, and V (Table 3).

The correlation coefficient showed a strong correlation between the BBS and sections II, III, IV, VI, and total score of the BESTest. In contrast, the correlation coefficient between the ABC-Brazil and BESTest was moderate only for section III and the total score, and for the other sections the correlations were poor (Table 4).

**Table 4.** Correlations between the BESTest with BBS and ABC-Brazil.

Section of the BESTest	BBS	ABC-Brazil
Section I – Biomechanical Constraints	0.62	0.36
Section II – Stability limits/ verticality	0.83	0.60
Section III – Anticipatory postural adjustments	0.70	0.29
Section IV – Postural responses	0.83	0.29
Section V – Sensory Orientation	0.62	0.38
Section VI – Stability in gait	0.82	0.35
**Total score**	0.78	0.59

BBS =Berg Balance Scale

**ABC-Brazil:** =Brazilian version of the Activities-specific Balance Confidence Scale.

## Discussion

The present study determined the validity and reliability of the BESTest translated to Brazilian Portuguese and adapted for individuals with hemiparesis after stroke. The interrater reliability for individuals with hemiparesis showed similar values to those reported for the original version by Horak et al.^14^. Although this study was conducted with individuals with various diseases, such as Parkinson's, vestibular dysfunction, peripheral neuropathy, and hip arthroplasty, it also found excellent interrater reliability (ICC=0.91) for the total score. Similarly, in the present study, the interrater reliability for sections I, III, and VI was excellent for participants with hemiparesis. Section VI, which evaluates stability during gait, showed excellent interrater reliability as in the results found by Jonsdottir and Cattaneo^22^, who assessed the reliability of the Dynamic Gait Index in individuals after stroke.

In our sample, sections II, IV, and V showed only moderate reliability, but ICC was >0.70 for all sections. In section IV, moderate reliability could be explained by the examiner's perception in relation to the patient's responses. Section V (remain standing on a stable and unstable surface with eyes open and closed) showed moderate reliability, unlike the original scale in which the timed results showed higher reliability^14^. In this section, most of the patients had scores of 3 (stable) or 2 (unstable), therefore the patients remained in the standing position for 30 seconds. The differences between two examiners may be due to the different perception of stability.

In patients with hemiparesis, the short version of the BESTest, known as the Mini-BESTest^15^, showed strong correlation with the BBS. Strong correlations between the BESTest and BBS are also reported for patients with Parkinson's disease^23^. Bergström et al.^24^ translated the Mini-BESTest to Swedish and validated it to individuals with Parkinson's disease and stroke. They also showed a strong correlation between the mini-BESTest and BBS for patients with stroke. The high correlation between the BESTest and the BBS can be explained because they evaluate the same construct and some items are similar in both scales although the scale steps differ. Despite this high correlation, in patients with hemiparesis, the BBS showed ceiling effects while the Mini-BESTest did not^15^.

In contrast, the ABC-Brazil showed a moderate correlation with the total score of the BESTest and section II, but a poor correlation with the isolated sections. This is consistent with previous studies that assess the correlation between performance measures and self-related measures^25^, and with the original BESTtest^14^as well as the Mini-BESTest administered to individuals with hemiparesis^15^. The BESTest is a test based on performance, and perceptions (as measured by the ABC) rarely predict the full variance of an actual performance^25^. Horak et al.^14^found a moderate correlation between the total score of the BESTest and ABC (r=0.68). The section II scores had the best correlation with the ABC (r=0.78) and section III, the worst correlation (r=0.41). This was similar to our study, which found the worst correlation in sections III and IV. In individuals with hemiparesis, the Mini-BESTest also correlated only moderately with the ABC^15^. Because the ABC evaluates perceived low and high-risk activities and low self-efficacy can result in avoiding such tasks, it is important to use it in conjunction with the BESTest and not as a replacement.

One limitation of our study was the small sample and the predominance of subjects with left hemiparesis (81%) and relatively good balance (score >43 on the BBS). However, the results of this study show that the Brazilian version of the BESTest demonstrated adequate reliability for the total score and when evaluated by sections. The small sample limits the generalizability of the findings, but the adaptations to the form of administration of the BESTest resulted in excellent concurrent validity with the BBS with the advantage of identifying what balance system is affected and then provide information for the development of a specific treatment. However, this scale is unable to identify which daily task presents the highest risk of falls and, if this is the objective of the assessment, it can be used in conjunction with the ABC, but not as a replacement. Additionally, other psychometric properties should be further investigated.
